# Phys-AbGAT: a physics-informed multi-task graph attention network for robust antibody-antigen binding affinity prediction

**DOI:** 10.1093/bioinformatics/btag669

**Published:** 2026-09-10

**Authors:** Yifei Yang, Linnan Xu, Junbiao Lu, Xiaoyan Li, Wei Zheng, Jianzhao Gao

**Affiliations:** School of Software, Nanchang University, Nanchang, Jiangxi 330047, China; Jiangxi Provincial Key Laboratory of Data Security Technology, Nanchang, Jiangxi 330031, China; School of Mathematical Sciences, AAIS and LPMC, Nankai University, Tianjin 300071, China; School of Mathematical Sciences, AAIS and LPMC, Nankai University, Tianjin 300071, China; Institute of Microbiology, Tianjin Centers for Disease Control and Prevention, Tianjin 300011, China; Tianjin Key Laboratory of Pathogenic Microbiology of Infectious Disease, Tianjin 300011, China; Key Laboratory of Prevention and Control of Major Diseases in the Population, Ministry of Education, Tianjin Medical University, Tianjin 300020, China; NITFID, School of Statistics and Data Science, AAIS, LPMC and KLMDASR, Nankai University, Tianjin 300071, China; School of Mathematical Sciences, AAIS and LPMC, Nankai University, Tianjin 300071, China

## Abstract

**Motivation:**

Accurate prediction of antibody-antigen binding affinity is essential for therapeutic antibody design. Existing methods often model the dissociation constant ($K_{D}$) and Gibbs free energy (ΔG) independently, overlooking their thermodynamic relationship and complex paratope-epitope spatial interactions.

**Results:**

We introduce Phys-AbGAT, a physics-informed multi-task graph attention network that represents the antibody-antigen interface as a spatial graph. It integrates ESM2 evolutionary embeddings with radial basis functions and employs a thermodynamically motivated auxiliary loss to encourage coupling between $\log_{10}\left(K_{D}\right)$ and ΔG. On an independent 42-complex benchmark, Phys-AbGAT achieved Pearson correlation coefficients of 0.569 for $\log_{10}\left(K_{D}\right)$ and 0.573 for ΔG. In matched-set comparisons, Phys-AbGAT achieved numerically lower RMSE and higher PCC than four baseline methods. The differences remained significant after Holm correction for MVSF-AB and PPA-Pred2, but not for AREA-AFFINITY or CSM-AB. Higher attention scores were assigned to several aromatic paratope residues, providing model-level hypotheses for structural inspection. These results support Phys-AbGAT as an interpretable multi-task geometric framework for joint prediction of two affinity-related quantities.

**Availability and implementation:**

Code and data are available at https://github.com/cliffgao/Phys-AbGAT.

## 1 Introduction

Antibodies (Abs), or immunoglobulins, constitute the primary molecules of the adaptive immune system, facilitating the high-affinity recognition and neutralization of diverse pathogens (Zhou *et al.* 2025). In the biopharmaceutical sector, monoclonal antibodies have established a transformative therapeutic paradigm for oncological, autoimmune, and infectious pathologies (Joubbi *et al.* 2025). A pivotal determinant of therapeutic efficacy is the binding affinity between the antibody and its cognate antigen (Ag), typically quantified by the equilibrium dissociation constant ($K_{D}$) or the associated change in Gibbs free energy (ΔG°) (Bandara *et al.* 2025). Thermodynamically, these parameters are intrinsically coupled via:


(#(1)) ΔG°=RTln(KDc°)=RTln(10)log10⁡(KDc°),

where $K_{D}$ is expressed in molarity, i.e. mol/L or M; c°=1 mol/L is the standard-state concentration; $R\;=\;1.987\times 10^{-3}\;\mathrm{kcal}/(\mathrm{mol}\cdot K)$; and T is the assay temperature in kelvin. The ratio $K_{D}/c{}^{\circ}$ is dimensionless, and ΔG° is reported in kcal/mol. For the base-10 target, $\log_{10}\left(K_{D}\right)$ denotes $\log_{10}\left(K_{D}/c{}^{\circ}\right)$ after $K_{D}$ is converted to molarity. For simplicity, ΔG° is denoted as ΔG throughout the remainder of this manuscript unless otherwise specified. Although experimental techniques such as Surface Plasmon Resonance (SPR) and Isothermal Titration Calorimetry (ITC) remain the gold standard methods for affinity characterization (Huang *et al.* 2025), these wet-lab procedures are labor-intensive, time-consuming, and resource-heavy, creating a bottleneck in high-throughput antibody screening and optimization. Consequently, there is an urgent demand for accurate computational methods to predict antibody-antigen binding affinity in silico (Yang *et al.* 2023).

Recent computational efforts to model antibody-antigen interactions have branched into sequence-based and structure-based methodologies. Sequence-centric approaches, such as PPA-Pred2 (Nikam *et al.* 2018), utilize functional classifications to estimate binding affinities across diverse protein complexes. More recently, frameworks like DG-Affinity (Yuan *et al.* 2023) and MVSF-AB (Li *et al.* 2025) have integrated sophisticated protein language models (e.g. TAPE (Rao *et al.* 2019), AbLang (Olsen *et al.* 2022), and ProteinBERT (Brandes *et al.* 2022)) with convolutional architectures (e.g. ConvNeXt (Liu *et al.* 2022)) to extract latent representations of the antibody-antigen interactions.

Compared with sequence-based methods, structure-based methods leverage the three-dimensional (3D) atomic coordinates of the complexes to capture the spatial nuances of the interaction. Tools such as CSM-AB (Myung *et al.* 2022) utilize graph-based signatures to model the interface and predict binding affinity (ΔG) using the Extra Trees algorithm. AREA-AFFINITY (Yang *et al.* 2023) utilizes interface descriptors to parameterize the binding surface.

Recently, more complex deep neural networks have been used in bioinformatics (Gao *et al.* 2016; 2021; Zeng *et al.* 2023). Graph neural networks (GNNs) have been increasingly adopted; for example, ProAffinity-GNN (Zhou *et al.* 2024) integrates protein language models with GNNs to predict $-log_{10}(K_{D})$, while ANTIPASTI (Michalewicz *et al.* 2024) utilizes normal mode correlation maps as inputs of a convolutional neural network to predict $\log_{10}\left(K_{D}\right)$ with high generalizability.

Despite these advancements, most existing models fail to fully encapsulate the complex spatial dependencies governing molecular recognition. Critically, contemporary predictors typically treat $\log_{10}\left(K_{D}\right)$ and ΔG as independent or interchangeable regression targets, providing no explicit inductive bias that links the two quantities through Equation (1). Consequently, independently predicted outputs may deviate from their thermodynamic relationship.

To address these deficits, we introduce *Phys-AbGAT* (*Phys*ics-informed *A*nti*b*ody-Antigen binding affinity predictor with multi-task *G*raph *AT*tention network). In this study, we present three primary methodological innovations that distinguish Phys-AbGAT from existing affinity predictors:

- Multi-task learning architecture: Phys-AbGAT simultaneously predicts the dissociation constant ($\log_{10}\left(K_{D}\right)$) and Gibbs free energy (ΔG), thereby addressing the limitations of single-target affinity predictors.
- Physics-informed regularization: We introduce a ratio-based auxiliary loss motivated by the thermodynamic relationship between $\log_{10}\left(K_{D}\right)$ and ΔG. This term acts as a soft inductive bias that encourages coupling between the two prediction tasks.
- Synergistic feature integration: Our framework synergizes high-dimensional evolutionary embeddings from the ESM2 protein language model with geometric graph attention networks (GATv2) and Radial Basis Function (RBF) edge encodings. This configuration allows for the precise capture of both latent sequence patterns and the complex spatial topologies intrinsic to the paratope-epitope interface.

Together, these components provide an integrated and biologically motivated framework for predicting antibody-antigen binding affinity.

## 2 Methods

### 2.1 Dataset curation and preprocessing

We retrieved the complete SAbDab (Dunbar *et al.* 2014) repository as of 17 November 2025. Structural files were parsed in Chothia numbering format, and entries lacking explicit binding affinity ($K_{D}$) or Gibbs free energy (ΔG) measurements were discarded. To support graph-based modeling, we excluded complexes consisting of a single sequence, resulting in a curated collection of antibody–antigen complexes for subsequent filtering and evaluation. For external validation, we utilized the Test42 benchmark (Guest *et al.* 2021), comprising 42 complexes with dual $\log_{10}\left(K_{D}\right)$ and ΔG annotations. Notably, the PDB entry “3RVW” was updated to its higher-resolution counterpart, “5VPG”, to ensure structural integrity.

### 2.2 Homology filtering and data partitioning

To reduce potential overlap with the independent Test42 benchmark, the SAbDab and Test42 sequences were pooled and clustered separately for the heavy, light, and antigen chains using the MMseqs2 (Hauser *et al.* 2016). Each complex was represented by a composite cluster signature ($H_{\mathrm{cluster}},\;L_{\mathrm{cluster}},{\;A}_{\mathrm{cluste}}$), with a dedicated missing-light-chain label. A SAbDab complex was removed when its signature exactly matched that of a Test42 complex. The remaining 632 complexes were randomly partitioned at a 4:1 ratio into 506 training and 126 validation complexes. The training and validation subsets were not constructed as mutually cluster-disjoint homology groups. Test42 was excluded from model development and hyperparameter selection. Detailed MMseqs2 parameters and multi-chain matching rules are provided in the Supplementary Methods, available as supplementary data at *Bioinformatics* online. The final data distribution is summarized in Table 1.

**Table 1:** Summary of the 632-complex development set and the independent Test42 benchmark.

Dataset	Complex	Heavy chains	Light chains	Antigen chains
Training	506	504	444	506
Validation	126	124	109	126
Test42	42	42	34	42

### 2.3 Graph representation of the interaction interface

**Figure 1:**
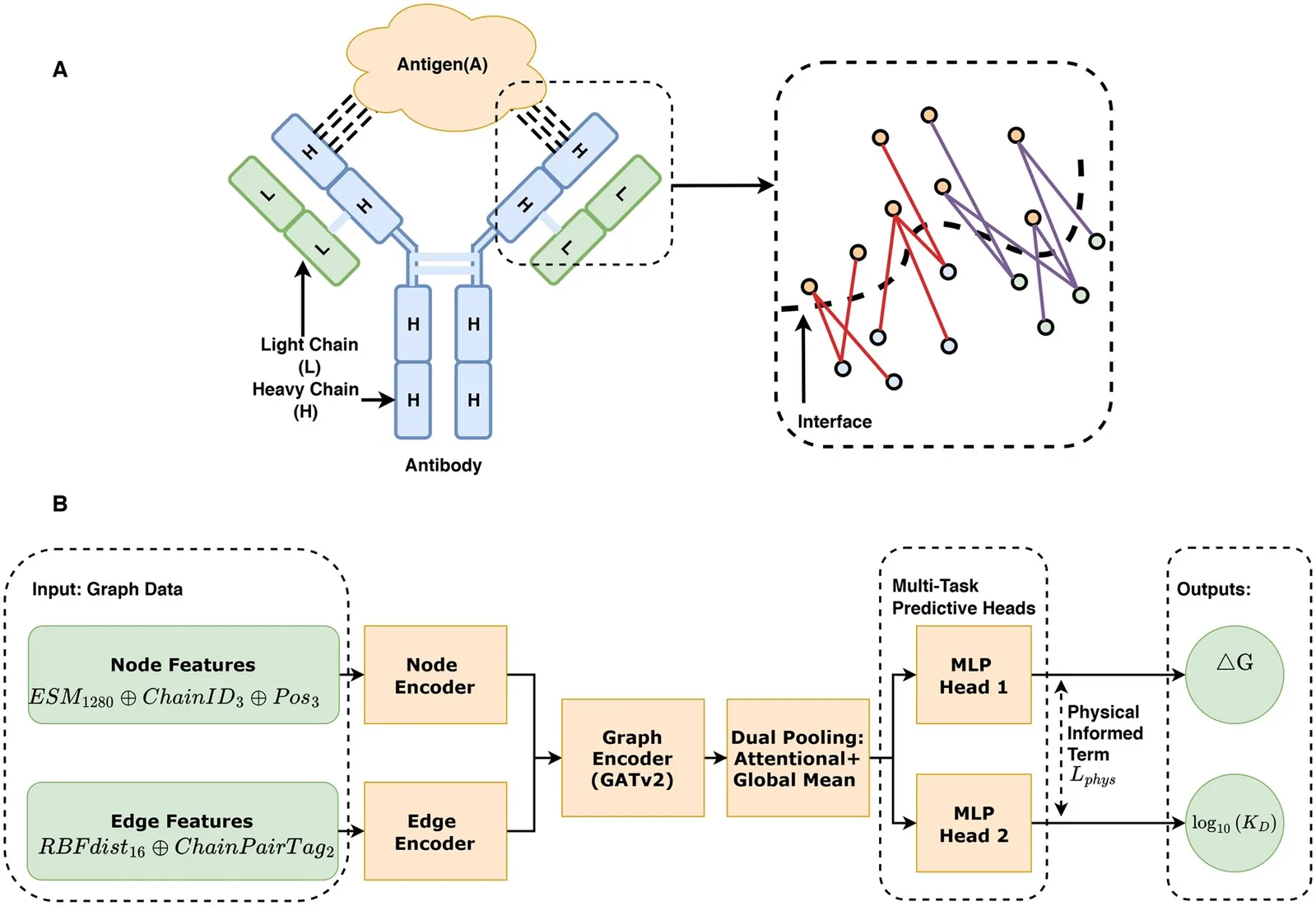
Architectural framework of Phys-AbGAT for physics-informed affinity prediction. (A) Graph-based parameterization of the antibody-antigen interface, modeling residues as nodes and spatial proximities as edges across heavy, light and antigen chains. (B) Phys-AbGAT workflow. The model uses 1286-dimensional node features and three-dimensional raw edge attributes consisting of a scalar inter-residue and two-dimensional chain-pair tag. The distance is expanded into 16 RBF distance components and concatenated with a two-dimensional chain-pair tag to form an 18-dimensional edge descriptor. Following feature encoding and GATv2-based graph propagation, a learnable attentional aggregation and global mean pooling were concatenated and layer-normalized to obtain the graph-level representation. Multi-task predictive heads simultaneously estimate $\log_{10}\left(K_{D}\right)$ and ΔG, with a thermodynamically motivated ratio-based regularization term ($L_{\mathrm{phys}}$) used to encourage coupling between the two tasks.

The antibody-antigen interface is modeled as an undirected graph G=(V,E). Nodes in the graph represent individual amino acid residues from the heavy (H), light (L), and antigen (A) chains. To capture the local chemical environment of the binding site, we utilized an interface filtering strategy: a residue is included in the graph only if its $C_{\beta }$ atom (or $C_{\alpha }$ for Glycine) lies within a 16.5Å Euclidean distance threshold of any residue on an opposing chain. In the absence of an antigen chain, a dummy graph consisting of a single zero-valued node without edges is generated. If the antigen is present but either the heavy or light chain is absent, a dummy node is inserted for the missing chain and subsequently removed by the 16.5 Å distance threshold. The graph is therefore constructed from the antigen-light or antigen-heavy interface, respectively. No sample in the training, validation, or Test42 datasets was represented by the single-node, zero-edge dummy graph. Figure 1A shows the graph representation of the interaction interface.

### 2.4 Architecture of Phys-AbGAT

The architecture of Phys-AbGAT is shown in Fig. 1B. *Node features:* Each node $v_{i}\in V$ is parameterized by a 1286-dimensional feature vector comprising: (i) Evolutionary embeddings (1280-D) extracted from the pretrained ESM2 language model (esm2_t33_650M_UR50D) (Lin *et al.* 2023); (ii) Chain identity (3-D one-hot encoded) to indicate whether the residue originates from the H, L, or A chain; and (iii) 3D Cartesian coordinates. We note that raw Cartesian coordinates are sensitive to global rotation and translation and therefore do not constitute a strictly SE(3)-invariant representation.

*Edge features*: For each edge $e_{ij}\in E$, the inter-residue distance $d_{ij}$ was expanded with 16 Gaussian radial basis function (RBF) components:


(#(2)) ϕk(dij)=exp⁡(-(dij-μk)22σ2),

where k=1, …, 16, $\mu_{k}=16(k-1)/15,$ and $2\sigma^{2}=1$. The RBF vector $\phi (d_{ij})\in R^{16},\;$was concatenated with a two-dimensional chain-pair tag $s_{ij}$, where [1, 0] denotes an antigen-heavy chain edge, and [0,1] an antigen-light chain edge. The RBF vector and chain-pair tag were concatenated to form an 18-dimensional descriptor:


(#(3)) eij=Wedge[ϕ(dij)||sij]+bedge.

Node features were projected to $d_{\mathrm{hid}}$ using a linear layer followed by LayerNorm and ELU, whereas the 18-dimentional edge descriptor was projected to $d_{\mathrm{hid}}\;$using a single linear layer.

*Graph attention propagation (GATv2)*: Phys-AbGAT updates node representations via L layers of GATv2 (Brody *et al.* 2021), utilizing a dynamic attention mechanism. The dynamic attention coefficient is defined as:


(#(4)) αij(l)=exp⁡(a⊤σ(Ws(l)hi(l−1)+Wt(l)hj(l−1)+We(l)eij))∑k∈N(i)⋃{i}exp⁡(a⊤σ(Ws(l)hi(l−1)+Wt(l)hk(l−1)+We(l)eik)),

where σ(·) denotes the LeakyReLU activation function, $e_{ij}$ denotes the encoded edge representation obtained from the RBF-expanded inter-residue distance and edge-type features. Node update with residual connection:


(#(5)) hi(l)=ELU(GSN(∑j∈N(i)∪{i}αij(l)Wt(l)hj(l−1)))+hi(l−1),

where $\alpha_{ij}^{\left(l\right)}$ is the attention weight assigned to the edge from node j to node i, $W_{t}^{\left(l\right)}$ is the learnable transformation applied to the neighboring node representation. GSN denotes GraphSizeNorm, defined as $\mathrm{GSN}(x_{i})=x_{i}/\sqrt{\left|V_{g}\right|}$, where $\left|V_{g}\right|$ is the number of nodes in graph containing node i. ELU denotes the exponential linear unit activation function.

*Multi-level global readout*: To aggregate node-level features into a graph-level embedding $z_{g}$, we implement a dual-pooling strategy. We combined attentional aggregation, which assigns learned weights to node representations, with global mean pooling, which summarizes the graph-wide representation. Attentional aggregation ($z_{\mathrm{att}}$) is defined:


(#(6)) zatt=∑i∈Vsoftmax(wgate⊤hi(L))hi(L).

Global mean pooling ($z_{\mathrm{mean}}$) is defined as:


(#(7)) zmean=1|V|∑i∈Vhi(L).

The attentional pooling vector $z_{\mathrm{att}}$ and global mean pooling vector $z_{\mathrm{mean}}$ were concatenated and further projected into a graph-level embedding:


(#(8)) zg=LayerNorm([zatt||zmean]),

where $z_{g}\in R^{2d_{\mathrm{hid}}}$. The final embedding $z_{g}$ is processed through parallel regression branches to simultaneously predict $\log_{10}\left(K_{D}\right)$ and ΔG.

*Physics-informed multi-task loss function:* Let $x_{i}=\log_{10}\left(K_{D}\right)$ and $y_{i}=\Delta G_{i}$ denote the two targets on their original scales. The targets were standardized using the training set statistics:


(#(9)) zx,i=xi-μxσx, zy,i=yi-μyσy.

The network outputs ${\hat{z}}_{x,i},\;$and ${\hat{z}}_{y,i}$, and the task losses were


(#(10)) LKD=1n∑i=1n(z^x,i-zx,i)2, LΔG=1n∑i=1n(z^y,i-zy,i)2.

The predictions were inverse-transformed before calculating the auxiliary term:


(#(11)) x^i=σxz^x,i+μx, y^i=σyz^y,i+μy,

The physics-informed term


(#(12)) Lphys=(1n)∑i=1n(y^i/x^i-yi/xi)2.

The total training objective was


(#(13)) Ltotal=(1-α)⋅LKD+α⋅LΔG+λphys⋅Lphys,

where α, and $\lambda_{\mathrm{phys}}$ are hyperparameters, $L_{\mathrm{phys}}$ was introduced as a thermodynamically motivated auxiliary regularizer. It encourages the predicted ratio to resemble the experimental ratio.

### 2.5 Model training and hyperparameter optimization

Model training was executed using the AdamW optimizer with an initial learning rate $l_{r}$. To ensure optimal convergence, we utilized the Optuna(Akiba *et al.* 2019) framework for automated hyperparameter optimization, selecting the final configuration based on the convergence of the objective loss function on the validation set via an early stopping strategy. The optimized parameters were determined as: learning rate $l_{r}=9.98\times 10^{-4}$, hidden dimension $d_{\mathrm{hid}}$=64, and loss weighting coefficients α=0.6 and $\lambda_{\mathrm{phys}}$=0.460. Further details on training and hyperparameter optimization are provided in the Supplementary Methods, available as supplementary data at *Bioinformatics* online.

Predictive performance was rigorously evaluated using Pearson’s correlation coefficient (PCC) to assess linear association and root mean squared error (RMSE) to quantify absolute prediction deviation. Both metrics were calculated across the experimental and predicted binding affinities. PCC and RMSE are defined as:


(#(14)) PCC=∑i=1n(y^i-y^¯)(yi-y¯)(∑i=1n(y^i-y^¯)2∑i=1n(yi-y¯)2),

(#(15)) RMSE=∑i=1n(y^i-yi)2n,

where n is the number of samples, $y_{i}$ is the actual antibody-antigen affinity value ($\log_{10}\left(K_{D}\right)$ or ΔG) and ${\hat{y}}_{i}$ is the predicted antibody-antigen affinity ($\log_{10}\hat{\left(K_{D}\right)}$ or $\hat{\Delta G}),\;\bar{y},\;\bar{\hat{y}}$ are the averages of the experimental and predicted binding affinities.

### 2.6 Benchmarking protocols and comparative analysis

To provide a comprehensive assessment of Phys-AbGAT, we benchmarked its performance against representative sequence-based methodologies (PPA-Pred2, MVSF-AB) and structure-based frameworks (CSM-AB, AREA-AFFINITY).

Baseline predictions were obtained using the official web services or publicly released source code. PPA-Pred2 predictions were obtained from the official web services using the antigen-antibody category. MVSF-AB was run locally using the authors’ publicly available source code. CSM-AB predictions were obtained from the official webserver API, whereas AREA-AFFINITY predictions were generated using the antibody-protein antigen module. A central objective of our benchmarking protocol was to reduce known training-test overlap. For tools with accessible training repositories, such as CSM-AB and AREA-AFFINITY, we performed a stringent cross-reference check with our independent test suite, retaining only non-redundant subsets (n = 13 and n = 16, respectively) for unbiased evaluation. Conversely, certain high-profile models were excluded from the comparison to maintain the integrity of our benchmarking: DG-Affinity was omitted due to the sustained unavailability of its implementation, and ANTIPASTI was excluded as the lack of transparent training metadata precluded a definitive assessment of structural overlap.

## 3 Results

### 3.1 Predictive performance and generalization capacity

**Figure 2:**
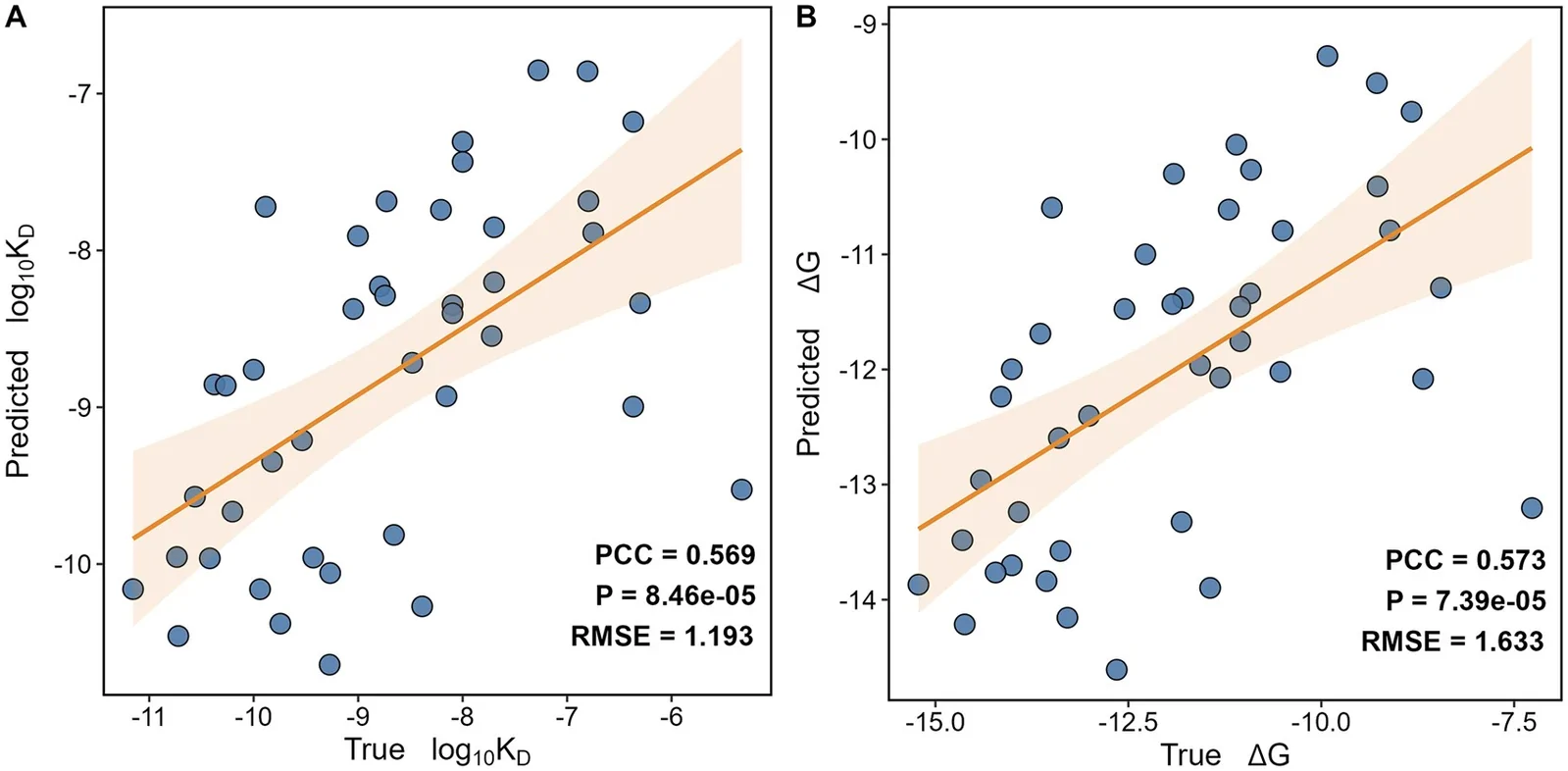
Correlation analysis of predicted versus experimental binding affinities on the independent test set Test42. Scatter plots illustrating the linear relationship for (A) $\log_{10}\left(K_{D}\right)$ and (B) ΔG. The solid orange line represents the linear regression fit, with the shaded region indicating the 95% confidence interval.

Phys-AbGAT demonstrated predictive accuracy on the independent Test42 benchmark using its multi-task geometric framework. As illustrated in Fig. 2, the model achieved moderate concordance with experimental values, yielding PCC values of 0.569 for $\log_{10}\left(K_{D}\right)$ (*P*-value: $8.46\times 10^{-5}$) and 0.573 for ΔG (*P*-value: $7.39\times 10^{-5}$). The corresponding RMSE values were 1.193 and 1.633, respectively.

The consistency observed between the validation set performance (PCC: 0.533 for $\log_{10}\left(K_{D}\right)$; PCC: 0.523 for ΔG) and these independent test results suggests that the model generalizes well to unseen antibody-antigen complexes. Comprehensive performance statistics, including RMSE, are documented in Supplementary Table S1, available as supplementary data at *Bioinformatics* online.

### 3.2 Comparative benchmarking against state-of-the-art methods

Based on the uniform method overview provided in Supplementary Table S2, available as supplementary data at *Bioinformatics* online, we benchmarked Phys-AbGAT against sequence- and structure-based predictors.

Because the four baselines returned predictions for different targets or subsets, each comparison was restricted to complexes with paired predictions from the baseline and Phys-AbGAT (Table 2). On the 42-complex PPA-Pred2 subset, Phys-AbGAT had an RMSE/PCC of 1.633/0.573, compared with 3.071/−0.097 for PPA-Pred2. On the 38-complex MVSF-AB subset, the corresponding values were 1.605/0.598 and 2.285/−0.049. On the 13-complex CSM-AB subset, they were 1.496/0.689 and 1.931/0.388. For $\log_{10}\left(K_{D}\right)\;$on the 16-complex AREA-AFFINITY subset, Phys-AbGAT had an RMSE/PCC of 1.148/0.530, compared with 1.258/0.439.

**Table 2:** Matched-set comparisons of Phys-AbGAT with baseline methods on identical complexes.

Baseline method^a^	*n*	Baseline		Phys-AbGAT	
		**RMSE** ↓	**PCC** ↑	**RMSE** ↓	**PCC** ↑
PPA-Pred2	42	3.071	−0.097	1.633*	0.573*
MVSF-AB^b^	38	2.285	−0.049	1.605*	0.598*
CSM-AB^c^	13	1.931	0.388	**1.496**	**0.689**
AREA-AFFINITY^d^	16	1.258	0.439	**1.148**	**0.530**

a Target Type of PPA-Pred2, MVSF-AB, and CSM-AB is ΔG; Target of AREA-AFFINITY is $\log_{10}\left(K_{D}\right)$. Bold type indicates the numerically better value in a row. * Holm-adjusted *P*-value < 0.05 in a one-sided paired permutation test; exact and adjusted *P*-values are given in Table S3, available as supplementary data at *Bioinformatics* online.

b Performance metrics for MVSF-AB were derived through local implementation in this study.

c Evaluated on a filtered test set that excludes structural overlap with the CSM-AB training dataset.

d Evaluated on a test subset excluding structural overlap with the AREA-AFFINITY training dataset.

One-sided paired permutation tests at the complex level showed significant differences after Holm correction for both metrics in the MVSF-AB and PPA-Pred2 comparisons. The AREA-AFFINITY and CSM-AB differences were not statistically significant. Because the rows contain different complexes and AREA-AFFINITY uses a different target, the values should be compared only within rows. Exact raw and adjusted *P*-values are reported in Supplementary Table S3, available as supplementary data at *Bioinformatics* online.

### 3.3 Contribution of protein language models

To evaluate the contribution of pretrained protein language models, we compared three backbones: ProteinBERT (Brandes *et al.* 2022), ProtT5 (Elnaggar *et al.* 2022), and ESM2 (Lin *et al.* 2023) on two affinity-related regression targets ($\log_{10}\left(K_{D}\right)$ and ΔG), using RMSE and PCC as evaluation metrics.

Table 3 shows a consistent performance ranking across both endpoints: ProteinBERT performed worst, ProtT5 provided a clear improvement, and ESM2 (this work) achieved the best results. Specifically, for $\log_{10}\left(K_{D}\right)$ prediction, ESM2 reduced RMSE from 1.447 (ProteinBERT) to 1.327 and increased PCC from 0.400 to 0.533, with ProtT5 falling in between (RMSE 1.346, PCC 0.518). A similar trend was observed for ΔG prediction. These results suggest that stronger protein language representations translate into more accurate and better-correlated affinity estimates, and that our ESM2-based model yields the best validation performance among the three evaluated backbones.

**Table 3:** Impact of pretrained protein language models (PLMs) on predictive performance on the validation dataset.

Model	$\log_{10}\left(K_{D}\right)$		ΔG	
	RMSE↓	PCC↑	RMSE↓	PCC↑
ProteinBERT	1.447	0.400	1.962	0.412
ProtT5	1.346	0.518	1.871	0.509
ESM2 (Phys-AbGAT)	**1.327**	**0.533**	**1.827**	**0.523**

Bold values indicate the best performance for each metric.

### 3.4 Ablation study

To evaluate the individual contribution of each architectural component within Phys-AbGAT, we performed a systematic ablation study across both affinity-related tasks. Table 4 summarizes the numerical contribution of the evaluated model components. The Phys-AbGAT configuration achieved the lowest RMSE and highest PCC among the evaluated variants for both tasks.

**Table 4:** Systematic ablation study of the Phys-AbGAT architectural components on validation dataset.

Variant	$\log_{10}\left(K_{D}\right)$		ΔG	
	RMSE↓	PCC↑	RMSE↓	PCC↑
w/o $L_{\mathrm{phys}}$	1.364	0.515	1.858	0.514
Single-task $\log_{10}\left(K_{D}\right)$	1.373	0.470	− ^a^	− ^a^
Single-task ΔG	−^a^	− ^a^	1.845	0.494
w/o mean pool	1.434	0.427	1.939	0.431
w/o attentional pool	1.391	0.480	1.882	0.487
w/o chain-pair tags	1.414	0.451	1.921	0.455
w/o RBF edge encoding	1.439	0.433	1.938	0.443
with regression share	1.406	0.439	1.906	0.442
Phys-AbGAT	**1.327**	**0.533**	**1.827**	**0.523**

a Dash(-) indicates that the corresponding target was not predicted under the single-task setting.

Bold values indicate the best results.

On the validation set, removing $L_{\mathrm{phys}}$ changed the $\log_{10}\left(K_{D}\right)$ RMSE/PCC from 1.327/0.533 to 1.364/0.515 and the ΔG RMSE/PCC from 1.827/0.523 to 1.858/0.514. These were modest numerical differences; no inferential test was performed for these predictive metrics.

To assess thermodynamic inconsistency directly, we calculated $r_{i}=\hat{\Delta G_{i}}-RT_{i}ln(10)\hat{\log_{10}\left(K_{D,i}\right)}\;$for the 31 validation complexes with recorded experimental temperatures (Supplementary Table S4, available as supplementary data at *Bioinformatics* online). Phys-AbGAT had a residual MAE of 0.143 and RMSE of 0.213, whereas the model without $L_{\mathrm{phys}}$ yielded values of 0.120 and 0.183, respectively. Defining the paired difference as the full Phys-AbGAT model minus the model without $L_{\mathrm{phys}}$, the MAE difference was 0.023 (95% CI: −0.006 to 0.055; two-sided paired-permutation *P*-value: 0.160), and the RMSE difference was 0.030 kcal/mol (95% CI: −0.009 to 0.065; *P*-value: 0.238). Neither difference was statistically significant. Thus, this post hoc analysis did not show that $L_{\mathrm{phys}}$ reduced direct thermodynamic inconsistency on this subset.

Removing the global mean pooling reduced PCC by 0.106 for $\log_{10}\left(K_{D}\right)$ and by 0.092 for ΔG. Removing attentional pooling reduced PCC by 0.053 and 0.036 for the two tasks, respectively, Excluding RBF edge encoding increased RMSE by 0.112 for $\log_{10}\left(K_{D}\right)$ and by 0.111 for ΔG. It indicates that high-dimensional geometric encodings are beneficial for capturing the non-linear spatial constraints of the paratope-epitope interface.

Notably, introducing an additional shared regression layer before the task-specific prediction heads increased RMSE and reduced PCC for both tasks, suggesting possible negative transfer from regression level parameter sharing. These results indicate that, although $\log_{10}\left(K_{D}\right)$ and ΔG are thermodynamically related, task-specific regression branches may better accommodate their distinct numerical scales.

### 3.5 Attention-based interface analysis

Our model achieves high interpretability through a dual-level attention mechanism that quantifies the contribution of specific residues and interactions to binding affinity. Edge importance is determined by averaging the dynamic attention weights across all GATv2 layers and heads, effectively mapping the key spatial contacts essential for complex stability. Concurrently, an attentional aggregation layer utilizes a gating network and softmax normalization to assign importance scores to individual nodes, highlighting functional hot spots such as epitope cores that drive the final affinity prediction. These normalized node and edge weights enable the 3D visualization of high-contribution regions on the antibody-antigen interface.

**Figure 3:**
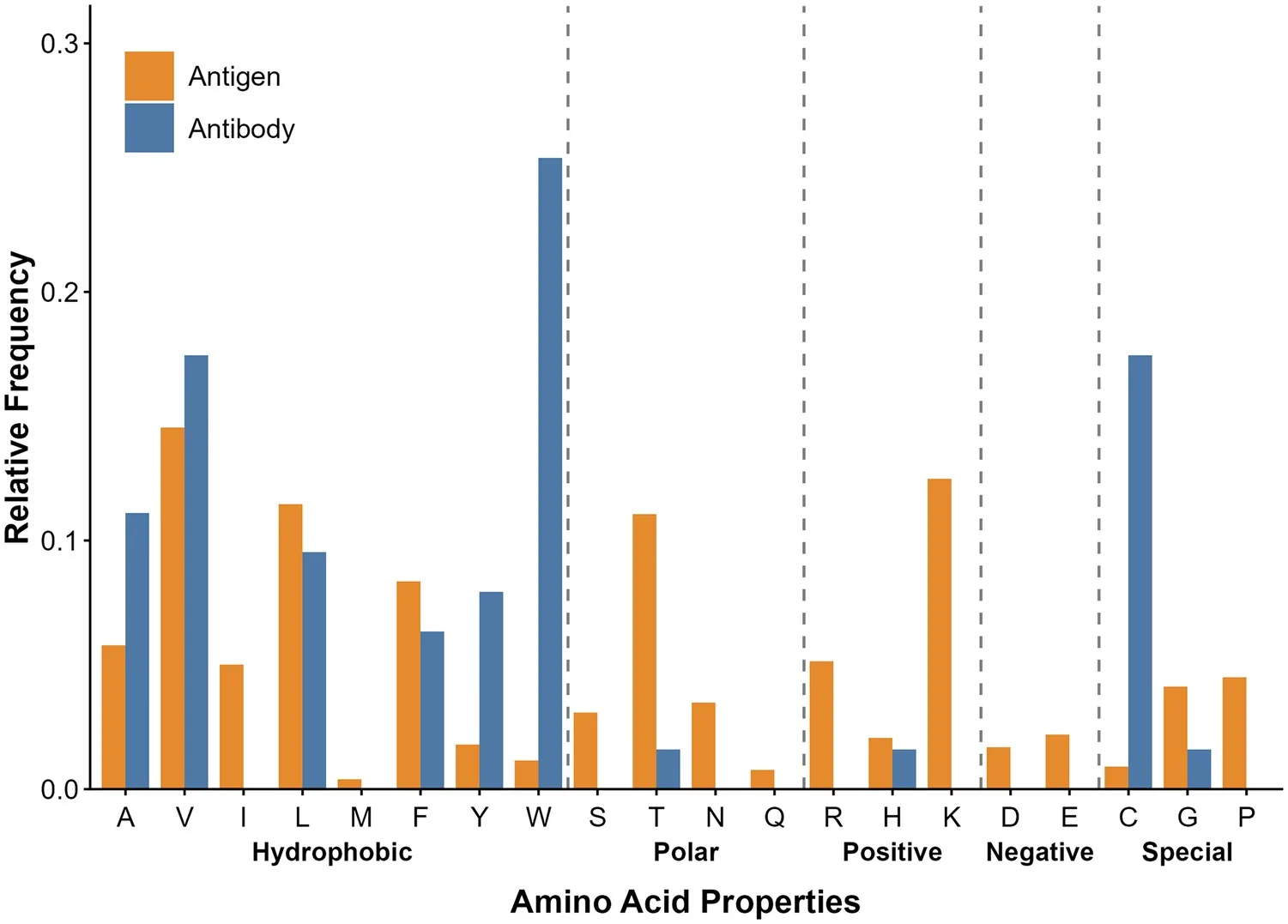
Physicochemical propensities of high-attention informative interface residues. Relative frequencies of selected interface residues on the antigen (orange) and antibody (blue) sides, grouped by physicochemical properties.

Interface residue composition analysis (Fig. 3) suggests divergent physicochemical signatures between the two binding partners. Antigen-side epitope residues were characterized by hydrophobic residues including Val(V) and Leu(L), together with aromatic Phe (F) accompanied by polar/positively charged residues such as Thr(T), Lys(K) and Arg(R). By contrast, antibody-side paratope residues were dominated by Trp(W), Cys(C), and Val(V). The Trp-rich profile may provide aromatic surfaces compatible with π-mediated recognition.

**Figure 4:**
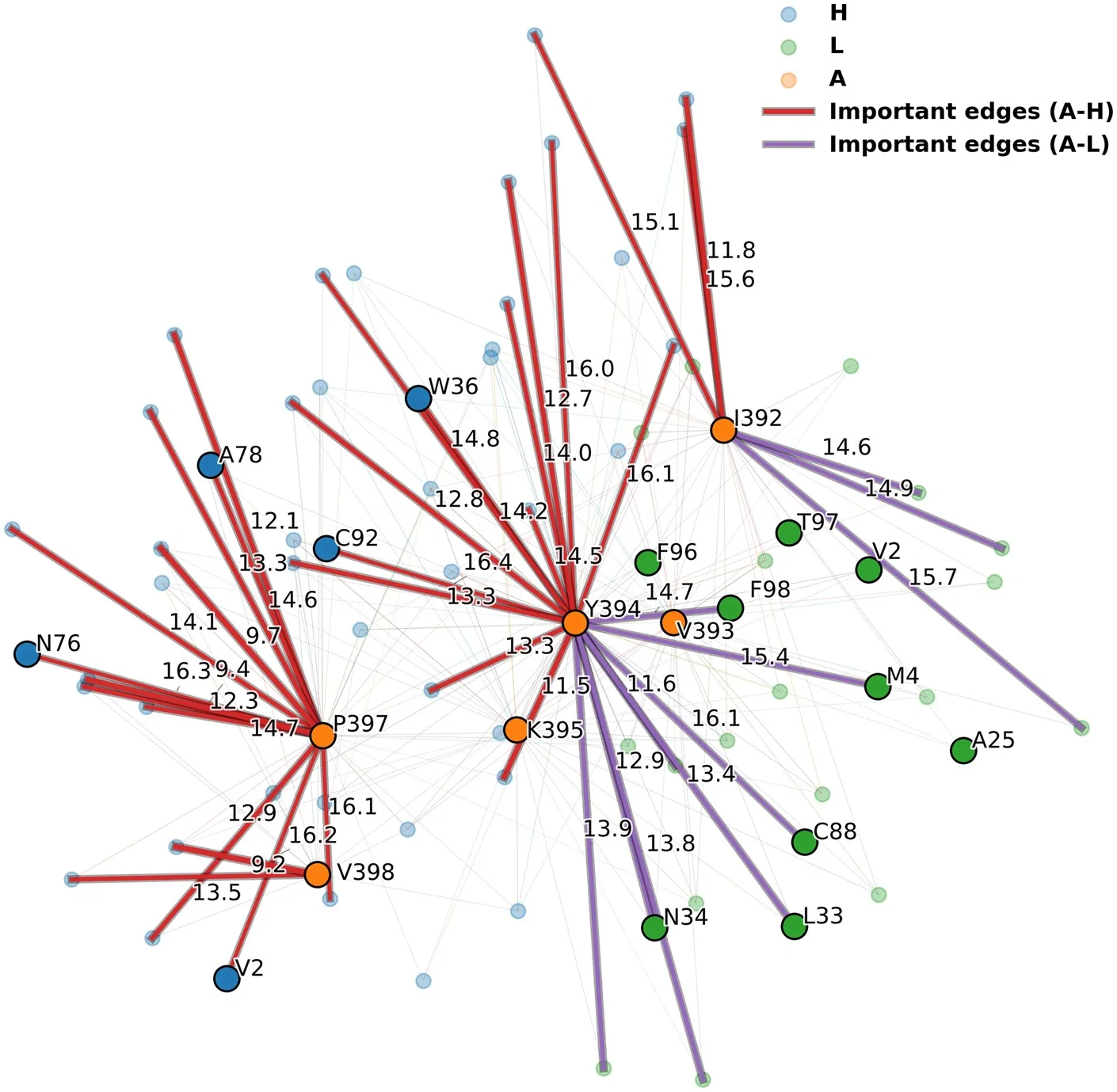
Attention mapping for PDB: 6BB4. Visualization of high-contribution interfacial residues (nodes) and residue level interactions (edges) prioritized by the attention mechanism. H, Heavy chain; L, light chain; A: antigen chain.

A representative structural case study of the antibody-antigen complex (PDB ID: 6BB4) underscores the interpretability of our framework. As illustrated in Fig. 4, residues and interactions prioritized by the model’s attention mechanism are highlighted, with critical interfacial edges depicted in red and high-contribution nodes indicated by increased marker size. Notably, the model identified long-range residue-level spatial associations within the 12.5–16.5 Å range. Although these distances, calculated between residue $C_{\beta }$ atoms (or $C_{\alpha }$ for Glycine), exceed the conventional thresholds of primary chemical bonding, they likely encapsulate longer-range spatial associations and global geometric constraints. These distal features appear to establish the essential conformational scaffold required for high-precision paratope–epitope docking and the subsequent optimization of binding affinity.

## 4 Discussion

The development of Phys-AbGAT marks a shift from purely data-driven black-box models toward physics-informed architectures in computational immunology. By synergizing ESM2-derived evolutionary insights with geometric attention mechanisms, our framework addresses the persistent challenge of accurately quantifying antibody-antigen binding affinity.

Phys-AbGAT jointly predicts $\log_{10}\left(K_{D}\right)$ and ΔG and uses a ratio-based auxiliary regularizer to couple the two tasks. Removing $L_{\mathrm{phys}}$ produced modest numerical changes in validation RMSE and PCC. However, on the 31 temperature-annotated validation complexes, the direct residual analysis did not show a significant reduction in thermodynamic inconsistency relative to the model without$\;L_{\mathrm{phys}}$. The auxiliary term should therefore be interpreted as a thermodynamically motivated soft regularizer, not as a hard constraint, and the predictive differences cannot be attributed specifically to improved thermodynamic consistency.

To ensure the integrity of our benchmarking, we implemented a redundancy controlled evaluation protocol. While this resulted in smaller benchmark subsets for CSM-AB (*n* = 13) and AREA-AFFINITY (*n* = 16), this was a deliberate consequence of our stringent redundancy filtering designed to reduce known structural overlap between training and testing distributions. Restricting the CSM-AB and AREA-AFFINITY comparisons to non-overlapping subsets reduced their matched sample sizes to 13 and 16. The resulting estimates are less affected by known training–test overlap, but their uncertainty is substantial. The nonsignificant results should not be interpreted as evidence of equivalence between methods.

The interpretability of Phys-AbGAT provides a quantitative bridge between geometric deep learning and molecular biophysics. The enrichment of aromatic residues like Tryptophan in high-attention paratope regions aligns with the hot spot theory of protein interfaces. Furthermore, the Phys-AbGAT’s sensitivity to long-range contacts (12.5–16.5 Å) suggests it captures essential longer-range spatial associations and global scaffolding context that precede stable complex formation. Additional experiments using five independent random seeds showed small standard deviations across validation and Test42 metrics, suggesting that Phys-AbGAT is relatively robust to random initialization (Supplementary Table S5, available as supplementary data at *Bioinformatics* online).

Several limitations should be noted. First, antibody-antigen complexes with reliable structures and experimentally measured affinities remain scarce; after redundancy filtering, our training/validation sets included 506/126 complexes, and the independent Test42 set included 42 complexes. Second, Phys-AbGAT builds interface graphs from 3D coordinates and distance-based edge features, so predictions may depend on structure quality, including missing residues, loop conformations, side-chain placement, and complex conformational state. Moreover, raw Cartesian coordinates in node features are not explicitly rotation- or translation-invariant, potentially limiting geometric robustness. Third, some non-redundant benchmark subsets for structure-based baselines were small after removing structural overlap, which should temper interpretation of comparative performance. Future work should develop larger standardized affinity datasets, assess structure quality explicitly, incorporate condition metadata, including temperature, pH, buffer composition, and ionic strength, and explore distance-based, local-frame, or SE (3)-equivariant representations. Fourth, sample-specific experimental temperatures were available for only 31 of the 126 validation complexes, limiting the statistical power and generalizability of the direct thermodynamic-residual analysis. Moreover, the implemented ratio-based regularizer can be sensitive when its denominator approaches zero and did not produce a statistically detectable reduction in the direct residual on this subset. Future work should collect more complete experimental-condition metadata and evaluate direct residual-based objectives that avoid division.

In summary, Phys-AbGAT provides a physics-informed graph framework for antibody-antigen affinity prediction by combining residue-level sequence representations, interface geometry, and thermodynamic regularization. The model offers a useful and interpretable basis for future development of structure-aware antibody screening and optimization.

## Supplementary Material

btag669_Supplementary_Data

## Data Availability

The code and data of Phys-AbGAT are freely available at https://github.com/cliffgao/Phys-AbGAT. Data is available at https://doi.org/10.5281/zenodo.18426407.
