# Supplementary material for: Phys-AbGAT: a physics-informed multi-task graph attention network for robust antibody-antigen binding affinity prediction
Source: Bioinformatics. 2026 Sep 10;42(9):btag669. doi: 10.1093/bioinformatics/btag669 (PMC13616043; doi:10.1093/bioinformatics/btag669)
Supplement: Supplementary file 1 [file btag669_supplementary_data.docx]

# Supplementary Material for **Phys-AbGAT: A Physics-Informed Multi-Task Graph Attention Network for Robust Antibody-Antigen Binding Affinity Prediction**

## **Supplementary Methods**

**Redundancy removal and data set partitioning**

To remove sequence redundancy between the SAbDab dataset and the independent Test42 dataset, complexes from the two datasets were pooled, and their heavy-chain, light-chain, and antigen sequences were clustered separately using the MMseqs2 easy-cluster workflow. Heavy- and light-chain sequences were clustered with a minimum sequence identity of 95% (--min-seq-id 0.95), whereas antigen sequences were clustered with a minimum sequence identity of 40% (--min-seq-id 0.40). For all three chain types, a minimum query-sequence coverage of 80% was required (-c 0.8 --cov-mode 2), meaning that the aligned region covered at least 80% of the cluster-representative sequence.

Each antibody-antigen complex was subsequently represented by a composite cluster signature, ($H_{cluster}$, $L_{cluster}$, $A_{cluster}$), comprising the cluster identifiers assigned to its heavy chain, light chain, and antigen, respectively. Complexes lacking a light chain, such as nanobody complexes, were assigned a dedicated missing-light-chain label. A SAbDab complex was considered redundant with Test42 and removed if its composite cluster signature exactly matched that of any Test42 complex.

After Test42-based redundancy filtering, no retained SAbDab complex shared the same composite cluster signature with any Test42 complex. The remaining 632 SAbDab complexes were randomly partitioned at a 4:1 ratio into 506 training and 126 validation complexes. Test42 was not used for training, validation, hyperparameter selection, or other model-development decisions.

**Training details and reproducibility**

The model was implemented using PyTorch and PyTorch Geometric and trained as a multi-task graph neural network to jointly predict $\log_{10} (K_{D})$and $\Delta G$. Each node was represented by a 1,286-dimensional feature vector. Edge distances were expanded using 16 Gaussian radial basis functions with centers uniformly distributed between 0 and 16 and concatenated with two edge-source indicators. The graph encoder consisted of $L$=2 residual GATv2 layers with GraphSizeNorm, ELU activation, and a dropout probability of 0.2. Attention-based and global-mean graph representations were concatenated and passed to two task-specific regression heads. The $\log_{10} (K_{D})$and $\Delta G$ targets were independently standardized using means and standard deviations estimated exclusively from the training set, and the fitted scalers were retained for validation and inference. Training minimized the composite loss

$$L_{\mathrm{total}}=\left( 1-\alpha\right)\cdot L_{\mathrm{KD}}+\alpha\cdot L_{\Delta G}+\lambda_{\mathrm{phys}}\cdot L_{\mathrm{phys}}$$

where the task-specific losses were calculated in standardized target space ($L_{\mathrm{KD}}$ and $L_{\Delta G}$)and the physics-consistency term $L_{\mathrm{phys}}$was evaluated after inverse transformation. Hyperparameters were optimized using Optuna over 100 completed trials. The learning rate was sampled logarithmically from ${10}^{-5}$to${10}^{-3}$, the hidden dimension from {8,16,32,64,128}, the number of attention heads from {2,4}, $\alpha$ from {0.1, 0.2, …, 0.9}, and $\lambda_{\mathrm{phys}}$ continuously from 0 to 0.5. All trials used a batch size of 32 and the AdamW optimizer with a weight decay of ${10}^{-2}$. Training was limited to 300 epochs, gradients were clipped to a maximum norm of 0.5, and a ReduceLROnPlateau scheduler halved the learning rate after 10 epochs without improvement in the mean validation MSE. Early stopping was triggered after 20 consecutive epochs without an improvement in either the mean validation MSE or the mean Pearson correlation across the two tasks. At each epoch, predictions were transformed back to their original scales, and the model-selection objective was defined as $\frac{1}{2}(MSE_{KD}+MSE_{\Delta G})$. The optimization was performed on an NVIDIA RTX A6000 GPU.

The best configuration was obtained in Optuna Trial 48, with a learning rate of $l_{r}=9.97734\times{10}^{-4},$ a hidden dimension of 64, two attention heads, $\alpha$=0.6, and $\lambda_{\mathrm{phys}}$=0.460438. The loss-selected checkpoint from this trial was used as the final model, while the independent test set was reserved for final evaluation and was not involved in hyperparameter optimization or checkpoint selection.

**Table S1. Performance of Phys-AbGAT on validation and Test42 datasets.**

| Dataset | $\log_{10} (K_{D})$ | | $\Delta G$ | |
| --- | --- | --- | --- | --- |
|  | RMSE $\downarrow$ | PCC$\uparrow$ | RMSE $\downarrow$ | PCC$\uparrow$ |
| Validation | 1.327 | 0.533 | 1.827 | 0.523 |
| Test42 | 1.193 | 0.569 | 1.633 | 0.573 |

**Table S2. Uniform summary of compared and related antibody-antigen binding affinity prediction methods.**

| Method | Input | Features | Model | Target | Benchmark status |
| --- | --- | --- | --- | --- | --- |
| **ANTIPASTI** | 3D^a^ | Normal-mode correlation maps | CNN | $\log_{10} (K_{D})$ | Discussed only |
| **AREA-AFFINITY** | 3D | Interface and surface-area descriptors | Ensemble method | $\log_{10} (K_{D})$ | Matched subset |
| **CSM-AB** | 3D | Graph-based structural signatures | Extra Trees regression | $\Delta G$ | Matched subset |
| **DG-Affinity** | 1D^a^ | Protein language model representations | ConvNeXt | $f(K_{D})$^c^ | Discussed only |
| **MVSF-AB** | 1D | Multi-view sequence features and PLM^b^ features | CNN | $\Delta G$ | Benchmark |
| **Phys-AbGAT** | 1D+  3D | ESM2 embeddings, chain identity, interface graph, RBF edge features, physics-informed loss | Physics-informed multi-task GAT | $\log_{10} (K_{D})$ and $\Delta G$ | Proposed method |
| **PPA-Pred2** | 1D | Functional or sequence-derived descriptors | Deep learning | $\Delta G$ | Benchmark |

^a^3D: protein structure; 1D: protein sequence.

^b^PLM: protein language model-derived representations

^c^$f\left( K_{D} \right)=-\frac{1000y_{kd}/591.282}{(\log_{e} (10))/10}$,where $y_{kd}$ is the value from (Cruz et al., 2023)

**Table S3** Complex-level paired permutation tests for the matched-set comparisons

| Method^a^ | n | Measure | Raw P-value | Holm-adjusted P-value |
| --- | --- | --- | --- | --- |
| PPA-Pred2 | 42 | RMSE | 0.000150 | 0.001050 |
|  |  | PCC | 0.000740 | 0.003700 |
| MVSF-AB | 38 | RMSE | 0.000610 | 0.003660 |
|  |  | PCC | 0.000040 | 0.000320 |
| CSM-AB | 13 | RMSE | 0.105957 | 0.423828 |
|  |  | PCC | 0.128540 | 0.423828 |
| AREA-Affinity | 16 | RMSE | 0.323486 | 0.646973 |
|  |  | PCC | 0.369904 | 0.646973 |

^a^One-sided paired permutation tests were performed at the individual-complex level. For n=13, and n=16, exact tests enumerating all $2^{13}$and$2^{16}$possible label-swap permutations were used, respectively. For n=38, and n=42, P-values were estimated using 100,000 Monte Carlo label-swap permutations with a random seed 20260826. P-values were adjusted across all eight comparisons (four methods $\times$ two metrics) using the Holm procedure(Holm, 1979).

**Table S4** Direct thermodynamic residuals for Phys-AbGAT and the model without $L_{phys}$ on the temperature-annotated validation subset (n=31).

| Metric^a^ | Phys-AbGAT (95% CI)^b^ | Without $L_{phys}$  (95% CI) | Paired difference  (95% CI)^c^ | Two-sided P-value |
| --- | --- | --- | --- | --- |
| MAE | 0.143  (0.097, 0.206) | 0.120  (0.080, 0.175) | 0.023  (-0.006, 0.055) | 0.160 |
| RMSE | 0.213  (0.113, 0.317) | 0.183  (0.100, 0.272) | 0.030  (-0.009, 0.065) | 0.238 |

^a^MAE and RMSE are reported in kcal/mol

^b^CI: confidence intervals. Model-specific confidence intervals and confidence intervals for the paired differences were calculated using a paired percentile bootstrap with 200,000 resamples.

^c^Paired difference means that Phys-AbGAT minus Without $L_{phys}$. Two-sided P-values were obtained by paired permutation tests with 1,000,000 within-complex swaps. For RMSE, the permutation was applied to the paired squared residuals. The paired difference was defined as Phys-AbGAT minus the model without $L_{phys}$; positive values therefore indicate a larger residual for Phys-AbGAT. Neither paired difference was statistically significant at the 0.05 level.

**Table S5. Phys-AbGAT performance across five random seeds on the validation and Test42 datasets.**

| Run^a^ | Validation | | | | Test42 | | | |
| --- | --- | --- | --- | --- | --- | --- | --- | --- |
|  | $\log_{10} (K_{D})$ | | $\Delta G$ | | $\log_{10} (K_{D})$ | | $\Delta G$ | |
|  | RMSE | PCC | RMSE | PCC | RMSE | PCC | RMSE | PCC |
| Random seed 1 | 1.341 | 0.516 | 1.829 | 0.514 | 1.192 | 0.561 | 1.634 | 0.564 |
| Random seed 2 | 1.340 | 0.538 | 1.817 | 0.542 | 1.203 | 0.543 | 1.632 | 0.565 |
| Random seed 3 | 1.339 | 0.529 | 1.808 | 0.535 | 1.170 | 0.590 | 1.562 | 0.613 |
| Random seed 4 | 1.359 | 0.518 | 1.835 | 0.524 | 1.237 | 0.580 | 1.694 | 0.584 |
| Random seed 5 | 1.331 | 0.528 | 1.792 | 0.535 | 1.161 | 0.588 | 1.606 | 0.590 |
| Mean | 1.342 | 0.526 | 1.816 | 0.530 | 1.193 | 0.572 | 1.626 | 0.583 |
| Standard deviation | 0.009 | 0.008 | 0.015 | 0.010 | 0.027 | 0.018 | 0.043 | 0.018 |
| This paper | 1.327 | 0.533 | 1.827 | 0.523 | 1.193 | 0.569 | 1.633 | 0.573 |

^a^All runs used identical data splits and hyperparameter settings. The five additional runs used seeds 414605, 262094, 291534, 159695, and 441168 respectively. The mean and population standard deviation were calculated across these five additional runs.

**References**

Cruz, V.L.*, et al.* Binding Affinity of Trastuzumab and Pertuzumab Monoclonal Antibodies to Extracellular HER2 Domain. *Int J Mol Sci* 2023;24(15):12031.

Holm, S. A simple sequentially rejective multiple test procedure. *Scandinavian journal of statistics* 1979;6(2):65-70.
